# Psychiatry and Politics in Pelotas, Brazil: The Equivocal Quality of Conduct Disorder and Related Diagnoses

**DOI:** 10.1111/j.1548-1387.2009.01073.x

**Published:** 2009-12

**Authors:** Dominique P Béhague

**Affiliations:** London School of Hygiene and Tropical Medicine

**Keywords:** [psychiatry, medicalization, politics, class conflict, conduct disorder]

## Abstract

The world-wide emergence of categories for diagnosing mental health problems in children and youth such as conduct disorder is often attributed to the globalization of a highly biomedical form of psychiatry. In Brazil, a small group of therapists are resisting biomedicalization by keeping psychodynamic traditions alive and aiming to transform psychotherapy into a resource for politicized youth empowerment. Nevertheless, clinical practices demonstrate an increased use of biomedical diagnoses and therapeutic routines. On the basis of fieldwork with therapists and teachers, and a nine-year-long ethnography of young people, this article explores the localized effects of these potentially contradictory developments. Results show that the growth of biomedical practices alongside politicized therapeutic approaches is not indicative of underlying ambiguities but has, rather, emerged from the purposefully equivocal nature of Brazilian social, medical, and professional life. The article uses this Brazilian case study to critically debate theories of medicalization in the anthropology of psychiatry.

Over the past two decades, the use of psychiatric diagnostic categories for diagnosing children and youth such as conduct disorder and attention deficit hyperactivity disorder (ADHD) has grown rapidly in North American and European psychiatry. The proliferation of these categories is an outgrowth of what some have described as a paradigm shift in psychiatry, from a more interpretive psychodynamic tradition to one that, imbedded in the standardized classification system of the *Diagnostic and Statistical Manual of Mental Disorders* (DSM-III/IV), purports to be context-free and easily reproducible (Anderson and Werry 1994; Luhrmann 2000). Sociologists, anthropologists, and a subset of psychiatrists have been quick to investigate the way this shift has shaped clinical and research practices in markedly reductionistic and individualizing ways (Conrad 2006; Lakoff 2000; Rafalovich 2005; Rhodes 2000; Timimi and Taylor 2004). Clinically, the creation of clear diagnostic entities based on standardized checklists of pathological behaviors has facilitated the widespread adoption of distinctly biomedical and pharmacological types of interventions. Epidemiologically, the ability to quantify disorders has enabled researchers to link mental health outcomes to genetic precursors and a series of biomedical and psychological risk factors, including poor parenting skills, maternal depression, prenatal exposure to alcohol and smoking, and low birth weight (e.g., Costello et al. 2007; Deater-Deckard et al. 2007; Edwards et al. 2007; Fryer et al. 2007; Nigg and Breslau 2007).

In most major cities in Brazil, disorders congruent with a more biomedical form of psychiatric practice are being increasingly researched and incorporated into clinical practice (e.g., Pastura et al. 2007; Polanczyk et al. 2007; Rohde et al. 1999). Even in Pelotas, a small town in southern Brazil where I have conducted several years of ethnographic research, the term *problemas de conduta,* or “conduct problems” was readily apparent in the lexicon of the psychiatrists, psychologists, and social workers I interviewed, and many were swayed by the precision that behavior-based ways of defining morbidity brought to their clinical interactions with young people (Béhague 2004). At the same time, and perhaps paradoxically, parts of the Brazilian ‘psi’ community (as the conglomerate of mental health care providers are termed locally) are actively resisting the biomedicalization of psychiatry, including the importation of the DSM-IV. Key psychiatrists, for example, have highlighted the detrimental impact that labeling young (often poor) people with “attention problems” or conduct disorder has on accentuating the stigma many of these youth already experience as marginalized members of society (Morihisa et al. 2007).

Resistance to the biomedicalization of psychiatry has been partially shaped by Brazil's recent deinstitutionalization and community-based psychiatry movements, which began flourishing with the health system reforms that were initiated in 1984 as part of wide-ranging postdictatorship governmental and political changes. Within certain subsegments of the psychiatric community in Pelotas, this era has witnessed the revival of longstanding interests in psychoanalysis, antipsychiatry philosophies, Marxist medical practice, and critical sociology. Several psychoanalytically trained therapists have committed themselves to changing what has often been criticized as an elitist and excessively introspective psychoanalytic tradition into a more popular and politicized form of clinical practice. Efforts to expand mental health services to a wider, more “popular” patient population by integrating psychiatry within preexisting school- and community-based initiatives have been met with enthusiasm by a wide range of professionals (Duarte 1999; Lessa 1997; Maurer Lane and Sawaia 2008; Silvestre de Paula et al. 2009). As some therapists argue, because much mental illness in Brazil is so clearly linked to socioeconomic inequities, therapy must not simply be sensitive to biographical, social, and economic determinants; it should be used as a tool for political emancipation (Béhague 2004).

From an anthropological point of view, the fact that multiple psychiatric ideologies coexist provides an invaluable analytical resource for conducting comparative analysis. Given the explicitly politicizing aims of an important subset of Brazilian psychiatrists, such a setting enables the exploration of how varied forms of medicalization, some more biomedical, others more explicitly social and “antibiomedicine,” both shape and possibly inhibit political consciousness raising and activity. The ethnographic work presented in this article is based on long-term fieldwork with therapists, school staff, local government officials, those involved in grass-roots movements, and young people themselves. Using an ethnographic life-course approach based on collection of both qualitative and quantitative data over a period of nine years with young people and their families, I give particular attention to ways processes of medicalization and politicization have come to be intertwined throughout the life course of young men and women. I use a comparative ethnographic framework to explore the specific instances in which psychiatric knowledge, values, and practices relating to the emergence of behavior-based disorders accrue power and authority. Alongside this, I explore how emerging psychiatric practices are, with time, empowering young people to embrace and further politicized struggles relating to class inequities and gender norms.

## Medicalization and the Anthropology of Psychiatry

Medical anthropologists have developed considerable insight into the question of how modern medicine garners power and authority. Steeped in a historically informed and nuanced understanding of the rise of biomedical institutions throughout the 19th and 20th centuries, these analyses, often implicitly and explicitly inspired by Michel Foucault's concept of “biopower,” show that medicine's authoritative practices often prevail even in the face of significant upheaval and resistance from within and outside of medicine. Specific accounts have demonstrated the centrality of power struggles over the use, dispersal, and salience of new diagnostic categories in accounting for the rise of biopower and for its effects in dampening local systems of meaning and “black-boxing” questions of existentialism. These effects are often attributed to biomedicine's tremendous institutional expansion, an expansion that has taken a particularly persuasive form in psychiatry over the course of the 20th century. Perhaps more so than any other area of biomedicine, emerging psychiatric practices have not only produced new clinical entities, but they have infiltrated into nonclinical institutions and domains, including the criminal justice system, formal education, and the workplace. In doing so, psychiatry has gained control over a number of nonmedical agents of medicalization, such as teachers and employers, thereby legitimately widening the institutional spaces through which the psychiatricization of the social realm is realized. Anthropologists, sociologists, and historians studying this process have shown that, among modern psychiatry's most powerful technologies, biological theories of causation have had a particularly profound depoliticizing effect in that they naturalize the social and dehumanize distress (e.g., Barret 1995; Birman and Costa 1994; Castel 1982; Conrad 2007; Ehrenberg 1998; Gaines and Hahn 1985; Good 1996; Kleinman 1988; Rose 1998; Sadowsky 1997).

The high degree of empirical consonance characterized by this substantial body of literature certainly represents the strength of an empirically rich understanding of modern medicine. However, some authors have also questioned whether such understandings should not, epistemologically speaking, be regarded with a healthy dose of criticism when applied to new settings and eras. (Frankenberg 1988). Perhaps more so than in other disciplines, anthropologists have argued for a constant vigilance of how our intellectual heritage shapes our own assumptions and ways of engaging with empirical observation (Stocking 1992). With regard to the intellectual origins of the (Anglophone) anthropology of psychiatry as we know it today, Allan Young has pointed to the developments of the 1970s as key. This era, characterized by a growing polarity between biomedical and psychodynamic psychiatry that fed into the DSM-III paradigm shift, was underpinned by the “decline of psychoanalysis in American academic psychiatry, the emergence of the antipsychiatry movement, … the publication of the English translation of Foucault's *Madness and Civilization* and the dawn of the golden age of psychopharmacology” (Young 2008, 299). Ethnographers who conducted their research during this era are understandably attracted to psychodynamic psychiatry and to the idea that biomedical psychiatric institutions, in most historical and social settings, are hegemonic, biologically reductionistic, and characterized by a tendency to blame and stigmatize individuals (Young 2008). However, underlying this attraction is a particular reading of Foucault's works that, as Ian Hacking has argued, has ceased being productive and now tends to reify futile debates about the “socially constructed” nature of illness (Hacking 1998, 1999).

These debates are key to the work of contemporary anthropologists of medicine and psychiatry, some of whom have devoted years of ethnographic and theoretical attention to the core question of how to move away from being structured by a polarity with social constructionistm on one end and scientific objectivism on the other (e.g., Lock 1991, 1993; Lock et al. 2000). One major challenge in moving away from such a polarization relates to the intense focus now awarded to the concept of “individual subjectivity.” Situated within an implicit social constructionist framework, the focus on subjectivity has sometimes been understood to mean, rather simplistically, as Biehl and colleagues argue, the study of those aspects of life that hegemonic discourses and structures allegedly silence—namely, “lived experience” and local meaning systems (Biehl et al. 2007). Social scientists taking up this agenda have often tilted the balance so heavily in this direction that what began as a crucial move to understand reality in a way that is meaningful to local actors has often been transformed into highly individualized accounts of personal experience, driven by the desire to give members of subordinated groups a “voice” (Desjarlais 1994; Littlewood 2003). As Butt has argued, many scholars disembed subjective statements from their broader context in order to validate their activist agenda, thereby limiting a more refined and empirical analysis of the production of health and illness (Butt 2002). Given this intellectual context, it is not surprising that ethnographers now struggle against an entrenched tendency to separate the study of “lived experiences” seen by those outside the field as the primary focus of ethnography—from the study of structural processes in the social production of pathology—aspects that are often relegated primarily to the work of historians, sociologists, and, more recently, social epidemiologists (Barret 1995).

By way of a small contribution to this debate, I suggest that the development of heuristic tools that challenge our own intellectual heritage and enable us to fully comprehend the social production of pathology is partially dependent on finding cases to study that represent exceptions to the workings of biopower. With regard to the anthropology of psychiatry, for example, we may focus on settings and historical moments in which the psychiatric deinstitutionalization movement, or certain aspects of it, succeeded or are succeeding in achieving its aims. To understand the conditions that enable this success does not preclude commitment to a critical approach. Crossley has shown, for example, how psychiatry's expanding process is intersecting not just with “counterdiscourses”: a variety of social movements have developed alongside antipsychiatry that, by introducing plurality, dynamism, and the potential for change, are contributing quite centrally to the way psychiatry is constituted (Crossley 1998, 2005). This dynamism means that some psychiatric practices are becoming politically and socially sensitized in ways that can be attributed to the legacy of the antipsychiatry movement, but that are probably quite distinct from the trappings that ultimately led to the movement's earlier failures in some countries (Béhague 2008).

## Methodology

For the focus on exceptions to acquire heuristic potential, the adoption of a comparative framework that enables both critical reflexivity and systematic macrocomparative analysis is essential. While comparative analysis entails a certain degree of categorization and, thus, reductionism, Fox rightly points out that units of analysis used to make comparisons “need not be accepted as discrete, homogeneous and stable entities” to be useful (Fox and Gingrich 2002:19). Moreover, the extent to which comparisons artificially construct reality depends less on the acts of categorizing and comparing per se, and more on the kinds of categories that are chosen and constructed, and on the way empirical findings are interpreted. Conceivably, should categories be chosen that reflect local realities and circumstances, comparative analysis could greatly enhance our understanding of how medicine permeates other social institutions and society at large to produce multiple forms of medicalization.

As noted above, the array of competing perspectives on the role psychiatry should play in Brazilian society lends itself to comparative analysis and the exploration of exceptions. The exceptions that are of particular interest in this analysis relate both to the Brazilian deinstitutionalization movement as a whole and to the way a minority of therapists and their young patients engaged with socially sensitive therapy and psi-induced politicization with particular fervor. Comparative analysis of these subgroups allowed me to discern patterns, to move beyond the description of individual accounts, and toward an understanding of how practices derive meaning from the way they are placed and circulated within society.

To this end, my research used a combined ethnographic and anthropologically informed epidemiological approach, imbedded in an ongoing 1982 epidemiological birth cohort study run by the Department of Social Medicine at the Federal University of Pelotas (Victora et al. 2003). The incorporation of a longitudinal ethnographic component in the 1982 cohort began in 1997, when the cohort youth were 15 years of age. At that time, a randomly selected subsample of 96 mother–child pairs was taken from the birth cohort and visited over several years by myself, an anthropological colleague based in Brazil (H. Gonçalves), and a team of four research assistants. This sample was chosen at random not with the aim of testing probabilistic hypotheses, but as a way of ensuring the inclusion of difficult-to-reach informants, and of inductively exploring whether patterns emerging in individual case-study analyses were salient for the larger sample of youth participating in the epidemiological cohort. Intensive periods of ethnographic fieldwork were conducted in 1997, 1999, 2000–01, and again in 2004–06. Several quantitative surveys have been conducted over the years, and some results from the 2001 survey with 1,033 youth, then 19 years of age, are included in this article. The longitudinal aspect of the study was critical for exploring how the relationship between medicalization and politicization is fluid if also socially patterned, based not simply on ideologies but on practices that change through time. To contextualize young peoples’ experiences, I also conducted interviews and participant-observation with upwards of 60 professionals working in an array of settings in which young people circulate, including schools, clinics, local government, and neighborhood associations.

## Medicalization and Politicization in Brazil: An Expanding Institutional and Ideological System

Although the utilization of private-sector psi providers by adults has been occurring since the 1960s in Brazil, the use of publicly funded psi services for young people is a new phenomenon, developing only in the past 10 to 15 years. Our quantitative results showed that levels of utilization of psi therapy among youth in Pelotas are quite high, comparable to that found in highly medicalized countries such as the United States. In our 2001 survey, conducted when the youth were 19 years of age, almost 30 percent had seen a psychiatrist or psychologist at some point in their lifetime. Although youth from the upper socioeconomic classes tended to use psi therapists in greater proportions than those from the lower socioeconomic class, use is still high among the lower class (defined as less than three minimum salaries per month): about one quarter of lower-class youth had seen a psi professional at some point in their lifetime, and of these, about one quarter were in the private sector (Behague et al. n.d.).

Such a high level of utilization is quite atypical for a middle-income country and no doubt reflects the presence of a combination of diverse influences. First and foremost is a significant increase in the population's access to specialist primary- and tertiary-level mental health care services over the past two decades. Since the implementation of health system reforms in the 1980s, Brazilians have been able to rely on a well-developed nationalized health service, with a geographically dispersed primary health care system providing reasonable quality care to the poor, and a vibrant private sector consisting of health insurance schemes and direct out-of-pocket payment. Several of the psi therapists I interviewed work in both public and private sectors and are using a sliding scale to charge patients from the lower to middle class a more accessible fee.

Also important in explaining high rates of psi utilization is the generalized populationwide acceptance of and demand for medicalized approaches to a number of diverse ailments, indicating a widespread culture of health care consumption irrespective of social class or income. Utilization of mental health services by adult women for “nerves” and problems relating to anxiety has become an entrenched norm over at least the past two decades, to the extent that mothers are likely to be a strong force driving medicalization among children and youth (Duarte 1986). Given this preexisting context, psi professionals have effectively been able to count on a ready-made institutional structure and cultural ethos of medicalization, which has greatly facilitated their efforts to generate interest in their services.

Equally if not more important in generating demand for psi services are therapists’ actual ideological and practical approaches to community-based psychiatry. For several public sector psychiatrists committed to the concepts of preventive medicine and popular psychiatry, therapeutic practice is defined in broad terms to include outreach into the community through engagement with schools and community-based organizations, alongside a wide range of activities to promote social participation and cohesion. These activities have been central for the dissemination of socially sensitive understandings of the causes of emotional distress. Teachers, parents, social workers, and youth I interviewed all tended to agree that most mental health problems are found among youth living in neighborhoods with high levels of poverty, where families not only suffer from deprivation but where youth are alienated from formal political processes and social tensions are seen to be widespread. For several therapists, approaching these cases with a view to supporting politicization meant increasing awareness among young patients (and youth more generally) of the economic and social injustices in Brazil society, a form of “consciousness raising” that many families were attracted to, and that teachers and school staff working with psychiatrists by and large welcomed and actively encouraged.

### The Relationship between Psi Utilization and (De)Politicization among Youth

Upon first encountering the developments described above, it seemed to me that psi therapists, and the teachers they worked through, were ignoring a profound contradiction in their professional practices. Despite a commitment to socially sensitive medicalization and clearly stated skepticism about the use of diagnostic entities emanating from the DSM-IV, categories such as conduct disorder and ADHD were not only discussed in programmatic planning meetings; they were actively diagnosed in clinical practice. When asked about this, several therapists stated that despite their ideological reservations, they found the child and adolescent diagnostic tools introduced by the DSM-IV to be a clear and useful way of raising awareness and focusing the attention of parents and teachers onto potential problem children. Some went so far as to cite a World Health Organization report as proof that in any population, it can be expected that about 20 percent of all young people will suffer from a “common mental disorder,” which, from the perspective of many of my informants, includes mild forms of ADHD and conduct disorder (WHO 2001). School staff I interviewed reproduced this concept, stating that about 20 percent of any given student population can be expected to need referral for psi therapy.

Our quantitative 2001 survey with the cohort youth confirmed the growing salience of DSM-IV–derived diagnostic categories. Among youth who were seen by a therapist, a bit over half used terms to describe their problems that were more psychodynamic in orientation, including traumatic life events (24%) and nerves–stress–anxiety and depression (31%). However, a not so insignificant proportion—about one third in total—were attributed to what I will term “behavioral disorders,” which include “aggression,” conduct problems, externalizing behaviors, and learning difficulties, including ADHD. Although few young people stay in therapy for periods of more than a few months, and the general pattern of use tends to be one of seeing multiple therapists for short durations at several different points over the course of several years, these diagnoses remained remarkably stable over the course of young people's early and late teen years.

To further compound the contradiction I thought typified psi therapists’ actions, I began to notice that behavioral diagnoses were attributed to lower-class youth in a highly systematic way by clinicians and school staff. This was confirmed in the epidemiological analysis, which showed that behavioral diagnoses tend to be more frequently diagnosed in the lower classes and among boys (Behague et al. nd), a pattern that is borne out in other settings as well (e.g. Maughan et al. 2004; Roberts et al. 2007). Such findings raise some key questions regarding the social dynamics that account for the distribution of diagnostic and therapeutic processes, as well as the apparent impact they may be having on stigmatizing and depoliticizing the working class. Historians and anthropologists have shown that societal concerns about the “deviant” behaviors of individuals from certain sub populations tend to arise in moments in which the elite feel threatened and resort to a number of techniques, such as the creation of particular social constructions that justify the medical control and containment of these individuals (Fisher 1985; Lock 1991).

In Brazil, anxieties about the need to better control the “underclass” are certainly prevalent, if implicit and intertwined with a concurrent humanitarian attitude that espouses empathy toward and integration of alienated youth (DaMatta 1993). With regard to the use of new psychiatric diagnoses and practices, however, I came to understand that the best way to describe phenomenon is not to say that therapists, teachers, and patients demonstrate unresolved ambiguities relating the use of behavioral categories, but rather that they are purposefully equivocal. As I will detail below, while behavioral diagnoses are increasingly in evidence in Pelotas, and while their use does initially correspond to a more reductionistic and individualizing way of approaching young patients, the therapy that ensued did not entail a straightforward process of depoliticization, particularly when viewed over time and when focusing on the production of exceptional circumstances. Rather, the referral pathways leading to therapy and the therapy itself held both depoliticizing and politicizing influences, both conflict-producing and conflict-resolving qualities. It is in part this dual quality that in some exceptional instances enabled socially sensitive psi therapy to have an eventual effect not only on fostering political consciousness but on nurturing the adoption of politicized practices as well.

### Politicization through Class Struggles: Young Boys

To understand how politicization emerges through exceptionally produced but socially contingent contexts, one must first understand normative patterns. In most instances, a boy who is “acting out”—that is, who is rowdy, disruptive in class, rebellious, and even marginally “aggressive”—is simply considered to be developing his masculinity and public persona. For many young men, engaging with the public realm by demonstrating “defiant” characteristics represents an important process of maturation, a way of garnering power, demonstrating assertiveness, and mobilizing a network of social allegiances. Because of these social norms, teachers, older peers, and even therapists appeared to have relatively high tolerance for such behaviors. When boys transgressed, the first port of call was most often simply disciplinary. Only repeated transgressions that were difficult to resolve within the classroom signaled a potential mental health problem. When referrals to the school psychologist were initiated, psi therapists were generally viewed by students, at least initially, as one and the same as other school staff, and the therapy itself as a mere extension of the school's disciplinary procedures.

For boys from families of low income, the tolerable threshold for disruptive behaviors tended to be lower than for middle- and upper-class boys. School staff referred lower-class youth for psi care more readily in part because they felt responsible for young men's future welfare and so felt compelled to give the lower classes additional scholastic and social support. Awareness of the social and economic determinants of emotional distress encouraged by psi professionals, as discussed at the onset of this article, is contributing to this development. In addition, disruptive behaviors are often statistically linked to low scholastic achievement in epidemiological studies, a pattern that affects lower-class boys more frequently and that has received considerable public attention (Tramontina et al. 2001).

The use of a lower threshold for identifying and referring problem cases was, however, also a reflection of prejudicial attitudes and assumptions on the part of school staff regarding the heightened “aggressive” and violent nature of lower-class youth. Several school staff expressed frustration with the difficulties they encountered in trying to discipline and teach youth who came to them “from shantytowns.” Often designated as *marginais* (socially marginalized and “vagabond” youth), these young people were said to have been reared in harsh social and economic conditions and, thus, to be particularly prone to aggressive outbursts. Several staff highlighted instances in which their attempts to maintain an empathetic approach based on nuanced interpretations of the causes of behavioral problems degenerated into conflict in the face of what they frequently described to be intimidating and threatening attitudes on the part of some young people. Exacerbating this trend was the fact that many lower-class youth feel socially awkward in school, and so tend to withdraw from interacting with teachers and wealthier school peers and avoid participating in optional school activities, making them a difficult group for school staff to interact with constructively.

Given the negative and, at times, antagonistic context in which such youth are identified for psi referral, it is no surprise that young lower-class boys experienced referrals in a disparaging, stigmatizing, and socially alienating way. As some youth explained, the referral itself is a personal affront to young men's personality and way of being and, thus, should be vehemently rejected. “Psychologists are here in the schools only to make money,” said one such boy. “They don't help, they just tell us what do to.” Initial therapeutic interactions that ensued were equally conflict ridden and challenging, such that some therapists felt forced to respond in a way that simply contained and controlled emotional outbursts. In these instances, therapy was indeed pathologizing and depoliticizing. Therapists described focusing first and foremost on the young patient's individual character traits and used techniques that transformed young men's demonstrations of “power” into “behaviors” that need assuaging. While some youth responded to therapy with continued defiance, others eventually accepted therapists’ interventions, allowing these to dampen their spirited view of the world. Paulo, for example, describes how the school therapist did not encourage him to engage in analytical discussions but simply “explained” to him what he should do:

[What did you and the psychologist talk about then? How long did you go for?] Oh, we would talk more about school, and how I didn't feel like studying, didn't feel like doing anything in class. I would just sit there and draw or talk to others. It was the teacher that sent me to go talk to the psychologist. Then I would go almost three times a week, we would set up a time, in the afternoon, during school hours. [And what would you talk about specifically?] Oh, they would tell me that without education, you can't do anything in life, tons of stuff like that … she would explain everything correctly, it helped some, things started changing a bit in school, I started studying more and calming down.

Though Paulo claimed to have started studying more, in actual fact, he eventually left school altogether. As these examples show, psi referrals and ensuing therapy often served to marginalize lower-class youth from schooling and from engaging in politicizing discussions and practices. It is important to highlight, however, that the depoliticizing processes imbedded in these psi interactions emerged not only because of, or even directly from, the psi professional's attitudes and actions, but rather from the way referrals to psi practitioners are used by teachers and other school staff to curtail young men's behaviors and address their social and scholastic difficulties. In other words, the depoliticizing aspects of psi medicalization emerged less from the clinic and more from the way psi knowledge and practice are used by members of the nonmedical world to communicate *social*—rather that strictly psychiatric—concerns relating to lower-class youth defiance.

This generalized pattern shows that teachers’ practices and clinicians’ interventions are linked to class-based stigma, depoliticization, and the continued alienation of the lower classes from normative society. Given my interest in understanding how exceptions to this pattern arise, I explicitly sought to analyze cases of young men who underwent therapy, but who diverged from this pattern, and who were, over time, empowered and politicized as a result of their therapeutic experiences. Of the nine boys in our sample who were referred to a therapist for a behavioral problem, seven lived on family incomes that are considered to be of low income; of these, four came to experience and contribute to an evolving form of socially sensitive therapy that produced positive politicizing effects. I compared these youth with those who continued to experience therapy as a stigmatizing process, as well as with a similar group of youth from our ethnographic sample who had not undergone psi therapy, but who nevertheless highlighted a number of emotional and psychological difficulties similar to those who used psi care.

The most overt experience that for these cases shared relates to the eventual development of a meaningful and personal understanding of social determinants of mental distress. Initially, most aspects of treatment for these youth were reductionistic and individualizing; therapists’ initial attention focused on the young person's individual experiences and behavioral problems as way of directly addressing the immediate problems at hand. Often, this included developing causal theories related to the *natureza,* or nature, of young people's “intellect” and “personality” within the context of multiple generations of poverty. Even so, these approaches did not exclude the subsequent, and at times concurrent, development of a more critical and political perspective. With time, the aim of the therapeutic interchange broadened to analyze a range of issues in subtle but in-depth ways. Discussions held in the psi sessions helped youth to create awareness not only of their own emotions but of how their lives are situated within larger socioeconomic contexts, which they came to believe accounted for their difficulties. This “critical consciousness,” as this awareness is often referred to (taking from the seminal and highly disseminated works of Paulo Freire [1990]), helped several of these young boys temper their reactions to what they described as an emotionally volatile and conflict-ridden school-based experience.

The developmental aspects of these changes cannot be underestimated. Flávio, for example, initially sought psi care for the emotional turmoil associated with the death of his brother and for what he described as adolescent-induced emotional “outbursts” of aggression—outbursts that became the source of many problems in school. After a year of therapy, Flávio explained that the changes ensuing from both his psi therapy and what he came to consider a “normal” process of maturation to adulthood were central contributions to the development of a sense of “critical consciousness.” As he stated,

It's not that you have to lose your charm (*graça*) when you grow up, but you must *tomar consciência*—become conscious of the fact that you have grown up, that you can't do this or that any more. So you calm down … If I have to have a serious conversation, I can do it without a problem. If there's a serious issue that needs to be discussed, such as when a friend of mine has a serious problem, I can help him, and he listens to me, he does what I advise him.

While therapy helped Flávio focus on “calming down” and outgrowing his outbursts, the critical consciousness he developed also gave him a space for reflecting on the larger societal reasons for his problems. On several occasions, Flávio readily acknowledged the importance of both his brother's death and his family's economic deprivation in making him feel “anxious.” So interwoven was his sense of emotional well-being with his socioeconomic situation that he often wondered if he and his family would ever achieve any degree of upward mobility. It was economic security, he explained, that would truly enable him to feel calmer and “more rested.” Therefore, for Flávio and other young men who underwent a more socially sensitive form of therapy, the cause of his distress was not understood to lie in any engrained character trait, or underlying physical problem, but rather with emotional difficulties linked to socioeconomic living conditions.

One may suspect that what Flavio and other youth like him experienced constitutes a conceptual reconfiguring of their behavior that simply scratched the surface of potential politicization and was never truly transformed in a way that reduces young people's sense of alienation from society, or that translates into sustained political participation. Indeed, anthropologists have shown that politicization in highly inequitable societies with relatively rigid class structures easily engenders forms of resistance that do not become revolutionary but remain, rather, part of the hegemonic institutional system (Scheper-Hughes 1992; Scott 1990). As Flavio himself insinuated, the urgings to engage with political consciousness may ultimately end in disappointment when confronted with the limitations of an intensely classist society.

However, there were clear indications of personal and structural transformation in what these young boys experienced. Over time, Flávio's interaction with psi therapy became powerful not because he continued to develop new-found narratives of politicization, but because he ultimately used psi therapy to address a number of practical issues relating to his social relationships in school and beyond. The more he, together with the therapist, interpreted his emotional turmoil and frustrations in a way that favored focusing on the social determinants of illness, the more he felt empowered to engage in social life, as well as in informal and formal political activity. Unlike many lower-class youth who feel socially awkward in school, Flávio actively pursued an informal leadership role, even though this often meant exposing himself to critique from peers, to increased probability of violence, and to living on the margins of multiple realities. Socially induced inner conflicts relating class relations such as these came to be a salient and productive part of Flávio's therapeutic focus. After two years of intermittent support from a psi therapist during times of increased social conflict, Flávio went so far as to become active in his school's club for student representation, an entity considered foreign and elitist by most lower-class youth.

These results suggest the evolution of a more complex, indeed cyclical, relationship between political consciousness-raising, medicalization, emotional distress, and political action. In several cases, initial debates regarding the behavioral disorder itself, whether “aggression,”“conduct problems,” or learning difficulties, became a tool through which political debates were nurtured and political activity stimulated. For many lower-class youth, political activity itself led to higher levels of mental distress, for it inevitably exposed them to relationships with peers from other socioeconomic strata and with whom conflicting power dynamics emerged. In several such cases, the relationships causing turmoil were explicitly pursued because youth, in analyzing the social sources of their emotional distress, actively placed themselves in a politically active role, instigating, for example, informal gatherings among acquaintances to discuss problems in school and neighborhood life. These youth often found themselves actively returning to psi therapy as a way of managing the difficulties arising from such situations, such that the use of psi therapy to problem-solve and assuage the stresses of classist institutionalized alienation was a shared occurrence among these youth.

The cyclical relationship between therapy and politicization also became a conduit for the transformation of personal politics into a more formal type of political consciousness and practice. As psi therapists helped these young men iron out social upheavals and related feelings of betrayal, criticism, and slander—“micropolitics” in an informal sense—they also stimulated them to feel more capable of engaging in formal political activities from which lower-class youth are generally alienated, including signing petitions, campaigning for student representation, voting in elections, participating in local youth chapters of political campaigns, and becoming active in local neighborhood organizations that advocate for the rights of shantytown dwellers.

Maurício's experiences demonstrate the expanding and time-dependent aspects of this cyclical politicization–medicalization relationship quite well. Maurício was initially sent to a psychologist by his mother when he was 13 years of age because of concerns relating to adolescent rebellion and “revolted” ways of being. At the time, he rejected the intervention, which he initially interpreted as a form of punishment. In later years, however, Maurício's reflections changed. As he described, the psychologist helped him to address his explosive nature and “think properly” about things:

Yes, I saw a psychologist, when I was younger, I think my parents thought I would become too rebellious, because in reality, they are not my real parents, so they were worried that I would become “revolted” (rebellious). [What would you do that made them worried?] Oh, I was a real pest, I was very “revolted” because at that time, I didn't think about things properly, I wasn't thinking. I mean today, I could be the same, I have it in me, I could be very rebellious if I start thinking about things [too much] … but now I know how to let it go a bit

Although Maurício came to accept the view that part of his problem lies in an inherent and ingrained way of being (“I have it in me, I could be rebellious always”), this did not preclude him from also considering the negative emotional influence of “thinking about things too much.” When asked to expand on the nature of these “things” and what exactly makes him “rebellious,” he mentioned aspects such as difficulty relating to his family, fear of violence in his neighborhood, and anxieties regarding future unemployment. In reflecting on his early experiences with therapy upon turning 19, Maurício did not feel his problems had dissipated; in fact, his personal circumstances remained much the same and had not objectively improved. However, he did feel he had learned to control his emotional reactions to his environment (to “let them go a bit”) and explained that, with the psychologist's help, he had stopped “reacting negatively” and in an emotionally volatile way.

Despite Maurício's initial focus on his own individual reactions and emotions, he developed, concurrently, a heightened politicized sense of the social and economic factors underlying his concerns with violence and unemployment. The dual ability to control emotional reactions and to understand their underlying social determinants was an essential stepping stone for Maurício's desire to find ways to avoid feeling alienated in the school setting and find ways of making his school, and society as a whole, more just. Rather than react to class tensions primarily with introversion and resentment, as many lower-class boys do, therapy allowed him address his emotional turmoil and focus on finding ways to gain legitimacy, currency, and clout in school. Alongside this, Maurício also began to initiate more generic debates regarding class, class-based conflicts, and economic inequities. With time he extended his concerns regarding the deleterious impact of societal injustice by analyzing the situation of lower-class peers, mobilizing on their behalf, and showing pride for his working-class background.

While it became clear that youth like Flávio and Maurício were engaging in politicization at both the level of consciousness raising and actual practice, there remains the important question of whether any of these interventions helped them to address the challenges that beset them. Might the final effect of such politicization simply be to leave youth content with their lower-class existence and prideful working-class identity? What I found was that, in these exceptional cases, therapy did indeed help youth tackle concrete problems such as scholastic achievement, both through direct applications of study skills and by helping youth manage feelings of alienation. Some were quite successful in finding ways to integrate into the school environment and temper the anxiety that often accompanied their attempts to do so. Seeking a better-quality life within the context of high levels of politicized discourse around working-class identities represented a delicate balancing act. While these boys aimed to increase their standard of living, a process that required them to gain at least partial proximity to institutional authority and upper-class values, they explicitly wanted to avoid becoming upwardly mobile in such a way that would alienate them from their home communities and persuade them, as some explained, “to buy into” the values of the upper class. For Flávio, becoming upwardly mobile was difficult not just because of the limited opportunities he experienced, but because he was forced to find ways to reconcile fear of critique from upper classes, with the fear of compromising one's working-class background and, through this, inadvertently discriminating against lower-class peers. Some therapists found ways to respond to this dilemma with considerable sensitivity, helping youth, through time, to understand and manage the emotions that arose from undertaking this balancing act.

For Maurício, the development of a multifaceted approach toward class mobility was most visible in the way his relationship with his mother unfolded throughout his teen years. Maurício's mother was very keen to see her children become upwardly mobile and focused heavily on education as the primary means of achieving such mobility. In her conversations with Maurício, she continually contrasted the opportunities her children now have (though still living in an urban shantytown) with the lack of opportunity she had experienced as a young person living in a very poor rural household. However, Maurício did not adopt her value system or comply with her wishes, and he continually rejected the merits of schooling, claiming that is it an institution “made for the rich” where the poor are treated like second-class citizens. In this and other ways, Maurício demonstrated a commonly ascribed to narrative of discontent with the upper classes and desire to keep “his” world separate from “theirs,” while also aspiring to ensuring fair treatment for all. As he stated,

I get along with everyone, rich, poor, women, men, gay guys, I treat all people as if they were the same, normally …. [so does that mean there is no difference between rich and poor?] Oh no, there is a difference; in my job (as a butcher's assistant), for example, I prefer to deal with poor people. They are more sincere, direct, and uncomplicated. Rich people are always criticizing, making problems, saying it could be better, more this way, more that way ….

Maurício's rejection of upper-class values and ways of being are typically used in everyday conversation by lower-class boys as a way of clearly demarcating themselves from poor youth who seek to become like the upper class. Maurício disdained boys who actively sought to mingle with the upper classes as part of their strategy to achieve upward mobility. For him, as for other youth like him, everyday class conflicts served to further underscore the inner conflicts that they experienced when thinking about the links between their emotions and class status. Although relatively innocuous when considered in isolation, at the level of the group, discussion regarding inequities and upper-class values could easily lead to inflammatory debates, heightened emotions, and the propensity toward violence. As for other lower-class boys exposed to these dynamics and committed to preserving their pride in being a member of the working class, Maurício's initial response was to avoid mingling with the upper class where possible. Yet with the support of therapy and over time, he found he was able to engage with youth of various socioeconomic backgrounds without compromising his lower-class value system. Once this inner conflict was resolved, Maurício immersed himself in school and his academic activities with greater enthusiasm and less apprehension. Such social abilities were key distinguishing characteristics for these young men for it helped them engage with the micropolitics of school life, with their studies, and with formal political processes in a way that upheld rather than negated their political beliefs and identities.

### Politicization through Gendered Class Struggles: Young Women

If the process of psi-influenced transformation enabled some boys to engage proactively with political debates and domains, how might socially sensitive medicalization play itself out in the case of young women, for whom access to the public world is generally restricted according to prevailing gender norms? In our sample, it emerged that a small group of girls (*N*= 6), all of them from the lower socioeconomic classes, were referred to a therapist for behavioral problems, despite the fact that this tends to not be a normative pattern in this setting. How did these young women's experiences, of both their problems and a potentially politicizing psi interaction, differ from those of the nine boys who were referred for therapy for similar problems? Is there a discernable pattern in the social and economic distribution of behavioral disorders and corresponding experiences according to gender, and how does this relate to the politics of class relations?

Because disruptive behaviors are considered more “masculine” in nature, any amount of behavioral defiance on the part of young women was not usually regarded as socially acceptable but was, rather, more readily linked to possible psychiatric morbidity. This meant that school staff were quicker to initiate the referral and diagnostic process for girls and that the psi process itself was more contested and politically expedient than for boys. It will be recalled that for boys, referrals to therapists were initially experienced as a normative if somewhat punitive process, and socially sensitive medicalization tended to ensue only after some time and only in some cases. For girls, in contrast, referrals were experienced as a logical, almost inevitable, consequence of their preexisting unconventional nature. These young women not only self-identified as “different from most girls,” particularly as it related to gender norms, but their behavioral outbursts—both before referral and once in the therapy—were explicitly motivated by a desire to contest larger social norms that relegate women to the private sphere of the household and restrict their entry into a form of public life that is generally reserved for men. Several girls, for example, highlighted their need for “freedom” from both the restraining socializing practices of their parents and the inequitable way teachers allow boys to wander the halls and playground indiscriminately, while actively reining girls in. While these young women actively shaped their identity based on what could be considered a localized form of youth “feminism,” they also acknowledged that their choices caused them considerable emotional turmoil.

One such girl, Ana, described herself as an “atypical girl,” for she both naturally achieved good grades at school (something most of her lower-class peers struggled with) and was constantly getting into trouble alongside her male peers. The daughter of rural migrants who had grown up in a shantytown, she explained that, unlike most girls with her background, she never shied away from new experiences, always sought to engage actively with the world outside her shantytown, and was naturally inclined to be forthright and “voice her opinions” when witnessing unjust situations. At the same time, she lamented that her “atypical nature” impacts negatively upon her emotional well-being. As she described, her active decision to visit friends in other parts of the city, to go shopping in the city center rather than in the safe confines of her shantytown, and to go to dance clubs unaccompanied by a boyfriend—behaviors that are usually considered inappropriate for young women, particularly if residing in a shantytown—exposes her to social criticism, potential violence, and social conflict. As she describes below, such conflicts are not only emotionally trying, but they also foster a strong sense of disappointment in government and the society in which she lives:

[There are lots of people on the streets that can do you harm ….] a politician was talking, it was close to the elections, and the politician had a *pivete* (young criminal, street youth), had him there because he was trying to show he would do something for the poor. Huh! My brother cried from anger, because they really should … stop making so much evil in the world …. Once I actually got in a fight with one of them, and I won, but it is dangerous, because sometimes they have knives. But even so, he looked at me like—I’m such a poor thing, how could you do that, like he was a victim, as if I were taking advantage of a child. But they are not children. I remember, I got so upset that day, my nerves attacked me and I vomited, the whole situation makes me vomit, politicians and street kids and all …

Given Ana's heightened behavioral and emotional reactions, it is certainly no wonder that she was sent several times to the school psychologist by her teachers for her “aggressive” behavior, something she clearly stated was unjust and precipitous. Why, she asked, were boys and not girls allowed to be defiant and “rowdy”? Though Ana questioned the motivations behind the referrals, and was critical in much the same way as her male counterparts, she was also not surprised to have been given a psi referral, and viewed it less as a disciplinary measure and more of an inevitable—if conflict-ridden—response on the part of the school to her atypical nature.

The conflicts young women like Ana experienced with school staff and, subsequently, with psi therapists upon being referred, were not only more intense than for young men, they were also consciously insisted upon and sustained by young women themselves. Margarete, for example, lives in a shantytown that is infamous for high levels of drug trafficking and violence. By the time she was 15 years of age, she had failed school two times, a pattern of school failure that is generally associated with young boys. Each year she failed, she was required to readapt to a new classroom of peers, and to new social and institutional dynamics, a process that only compounded her social and scholastic difficulties. After several years of struggling with school authorities, she switched schools to see whether she could improve in a new environment, but she continued to be identified as a “deviant” student.

When asked about the psi referrals she received, Margarete was quite negative and rejecting. While she acknowledged her difficulties, she firmly stated that she had failed so many times in school not because she was “agitated” or naturally got “bad grades,” which in her view would have been a reflection of her emotional state and intellectual abilities. Rather, she actively chose to spend time on the streets and away from school, an attitude more frequently found among boys:

[And how are things in school these days?] Well, I’m in the first year of the second level (high school)—for the third time. Because I was studying in one school that was near the center, near the *calçadao*[place frequented by many youth, usually young men], and so I would just end up going there to talk. So I failed basically because I was never there. I failed from absence, not from “lack of grades.” I’ve always found ways of cheating, but still I ended up failing overall, even though that has often helped quite a bit. My mother doesn't know, she thinks it's because of grades …

Highlighting the fact that the school psychologist inappropriately pointed toward Margarete's “behavior” problems as the cause of her academic failure, Margarete rejected all recommendations put forth by the school's psychologist and prioritized “getting by in school” through cheating. It would be incorrect to state that she did not value education; rather, Margaret clearly stated she felt unjustly “singled out” and shunned by school staff, and that this, rather than a dislike of studying, was at the core of her own rejecting behaviors. Like other girls identified with behavioral disorders, Margaret was unique in that she was not only aware that such behaviors were typically masculine but took pride in demonstrating her defiance of school authority throughout her school life even if, on several occasions, she admitted to feeling sad that her lack of conformity had made her social life as a young teen less predictable and jovial.

Although a volatile and potentially alienating process, the interactions that Margaret and girls like her had with the psi interlude also differed from their male counterparts in the degree to which the encounter was precipitated by and enabled explicitly political attitudes. Both young men and women initially rejected the referral. However, young women diagnosed with behavioral problems were clearly politicized from the outset, something they had been practicing for many years and was at the heart of their behavioral outbursts. It will be recalled that politicization in young men was nurtured throughout the course of medicalization and their reactions to school-based experiences. Initially, at least, there was nothing explicitly politicized about these young men attitudes toward everyday life. In contrast, for young women who had developed as self-referentially social rebels from a young age, psi therapy was initiated with a clear set of political motivations.

In the case of Margaret, for example, the behaviors that led to her psi referral were congruent with her unconventional approach to gender norms as a whole. In many ways, Margarete actively and explicitly adopted a “male pattern.” She was not only continuously rowdy in class but had dozens of brief romantic encounters with boys and did not worry about the rumors that her “sexual laxity” was likely to instigate. Instead of becoming heavily involved in “gossiping” with other girls, she avoided intimate friendships with most girls, explaining that girls are “competitive, generally untrustworthy, and prone to destructive envy.” She confided in only a few select girls her own age and said she preferred, rather, to both date and befriend boys, a position that contradicts local gender norms. Although Margarete knew many of her behaviors were viewed by school staff as inappropriate and even as “signs of emotional distress,” she consciously persisted in implementing her choices and doing so in a public way. Therefore, the very act of being identified as “behaviorally disruptive” through the psi referral served the important political function of repudiating segregationist gender-based values.

Given the conflict-ridden nature of these young women's experiences, one might expect psi-induced transformation, when and if it did occur, to be even more punctuated and gradual than it was for young men. I found, however, the converse to be true. Therapeutic politicization was enabled not through a subtle nurturing process as it was for boys, but rather through a process of repeated contestation, often played out by young women with the therapist as representative of institutional authority. The mechanisms accounting for this transformation were similar to those described above for boys; that is, politicized consciousness raising both within and outside of the clinical context often aroused not just political action, but also heightened emotional anxieties relating to the politics of class relations, a process that itself led to the search for more psi therapy. In the case of young women, however, this cycle was considerably more intense, debate ridden, and emotionally draining. These girls explicitly stated that their problems with nerves and stress resulted from the struggle of living in poverty and of engaging with an unfair world in a way that is explicitly confrontational. Though the clinic provided a safer and more supportive environment than is found in society as a whole, the fact that the therapeutic experience reproduced these politicized struggles led to both higher levels of mental distress and a continued desire for the therapy itself.

Interestingly, as therapy progressed, the focus of psi discussions shifted from the transgression of gender norms to the issue of economic inequities and how class-based discrimination accounts for emotional distress. In this way, preexisting politicization through localized feminism became an entryway for these young women to engage in politicized debates regarding economic inequities. To some extent, this shift provided these young women with some emotional relief and, with several outlets for political activity. By engaging with the analysis of class inequities, young women were effectively adopting a more socially applicable and salient political agenda, one that gives them more legitimacy, leadership, and expediency than that of arguing for gender equality, which in fact many lower-class men do not agree with. “Acting out,” then, was a calculated statement against not only the values of rigid gender demarcations, but of the upper class as well.

Eliane's experiences are particularly demonstrative of this more explicit transformative process. A young woman from a lower-class family living in one of Pelotas’ more isolated and infamous shantytowns, Eliane stated she was “different from most girls,” having chosen an active social life on the streets and taken the freedom to date several young boys irrespective of possible ensuing gossip and slander. By age 14, she had failed two years of schooling and was sent to a psychologist several times for “acting out” and “attention problems.” As Eliane stated, when we visited her in 1998,

I have a problem with memory, I can't retain it all, so I have to study right before the test, or else I fail. [Have you always had it?] Oh, yes, my whole life. I always had to go to a psychologist. [Was is the school psychologist?] No, it was a private sector psychologist. [And who pays for it?] My mother, it used to be three times a week. It was mostly when I was in fifth grade that I went more. It was like this: I would study and study and study, and then when the exam came, I would draw a blank. And so, if I could not remember, I would just cheat. [And these days, are you cheating?] When it's possible, you know, not always that easy to not get caught.

At the same time that Eliane accepted an interpretation of her problems based on her individual difficulties with “memory,” she was also quite critical of teachers and schooling, stating that although there are “some nice teachers, most of them just yell at you, they don't care at all about the actual students.” Another psychologist whom she visited when she turned 18 supported Eliane's inclination to widen her interpretation of the reasons for her scholastic difficulties. During this time, she developed a more refined view of her problems, explaining that her learning problems related not only to “memory blanks” during test taking, or even to “bad teachers,” but to the stresses of everyday life. As Eliane stated, “you often forget everything for school because you just start thinking about the problems we have,” referring specifically to the stresses that poverty and unemployment imposed upon her already strained relationship with her controlling mother. Indeed, Eliane spent a considerable amount of time discussing detailed aspects of finances and, cost of living, and how these contributed to her “stress” and “nervous disposition.” By young adulthood, Eliane's view of economic inequities had broadened considerably. In one of our encounters at that time, she quickly entered into a spontaneous and confrontational discussion of the injustices that “the poor” must endure:

I will not say that I am happy, no, because I’m not, helping my mom at home is hard, but I also don't like to just sit around. I am doing everything possible to try to get work, but it's really hard … you know, its the rich people that make us people poor, no really, I mean it. [why do you think that?] Because if we work for a rich person, you earn a tiny amount, a miserable amount, ok, so it's a minimum salary and a half, but you work like crazy, you do everything … then they tell you to do just one more little thing and then before you know it they are exploiting you … don't you go denying it! They want us poor people to be fucked, they are not interested in us. I think this way, because it is the truth. For now, it's ok because I am not *passando fome* (feeling hunger), thank god, but it's awful. There are people here (in the neighborhood) that are always asking, begging, and they are right, because they don't have food, they are right to ask.

As she matured and with the continued support of her psi therapy, Eliane's socially motivated, politicized attitude focused less on herself and more on generalized inappropriate understandings of young people. For example, she actively rejected commonly used (conservative) theories of the role of parenting in shaping youth criminal pathology and stated, instead, that the problem lies with “the government [that] does nothing to help people young people get jobs.” Quite radically, she continued by explaining that:

the government gives all their money to the retired, that is why young people steal, and that is why I sometimes say, if you have steal from people who have more, I’m not shy, I say it directly to the wealthy, take from those who have so much more. Think about it, today, what is the prison today? It is a house, a home [for the poor] because you have food, you can have visitors, you can have friends … there are people who are eating better in prison than out here ….

Though clearly contentious, an important consequence of discussions such as these was an increased acceptance of psi therapy itself. Indeed, Eliane's criticisms of the upper classes had initially been directed at any adult officially linked to authoritative institutions, and she had been quick to dismiss school therapists as part and parcel of the indifferent elite. With time, however, her opposition was targeted less at the psi therapists themselves and more at the school, which she critiqued for being a complex and inauspicious institutionalized environment that requires careful maneuvering on the part of the lower class. As she proceeded to discuss her experiences with the psi therapist, she developed a clear distinction between the stigmatizing practices of school staff who referred her for therapy and the more nuanced supportive practices of the psi therapists she came to know. Indeed, like Eliane, all the girls who underwent therapy for behavioral problems ultimately did not reject psi therapy wholeheartedly, as did some of their male counterparts, but accepted certain aspects of what psi therapy had to offer. Ana, for example, contested the school structure and gender norms, but she was also surprisingly accepting of the way the therapist could help students understand the challenges of the school environment, stating that while “the [school] director isn't worth anything—she just stays in her office and doesn't do anything, that's why the school is falling apart—the psychologists explained things about why things go wrong. Schools need psychologists because everything is chaos in schools now … [and] the young person is too agitated, very aggressive.”

The fact that these women used the therapeutic interactions to directly confront class conflicts also meant that they felt empowered to focus not only on their own well-being, but that of their communities and peers as well. Margarete, it will be recalled, was initially quite vocal about her dislike of the therapist she was referred to, but by the time we saw her at age 18, she was visiting the school psychologist once again, this time at her own instigation and without her mother's knowledge. With the psychologist's help, Margarete continued to work through the social and emotional consequences of her unconventional choices and behaviors. Though she accepted a need for therapy, Margarete never abandoned her political inclinations and attitudes and, if anything, in subsequent years demonstrated a refined sense of the importance of engaging in practical attempts to improve the community in which she lives. So much did her approach to her own psychological and social development change that she had even begun to consider clinical psychology as a professional option in life when we last visited her, at age 22.

Similarly, Eliane's attitude was not entrenched in a straightforward, alienating lower-class position, like some of her lower-class schoolmates, for she found ways to engage actively with her communities and schools. In her early 20s, Eliane became quite interested in local politics and the coming elections. She demonstrated deep skepticism of the mayor's promises to provide more jobs and argued that employment and health should be high on politicians’ list of priorities. Although Eliane had initially stated that she did not believe in the political process enough to register to vote—stating, for example, “the problem is no one thinks like the *povo*[the people, the populace], they all [politicians, upper class] think differently than we do”—she did eventually choose to take part in the elections.

For these unique and unconventional young women, socially sensitive psi therapy was both the product of, and a nurturing force for, politicization relating to the contestation of gender and class norms through micropolitics. The mere medicalizing process—the diagnosis, the referrals, the therapy—was politicizing in that it became key to rendering conflicting interactions and viewpoints poignantly visible, and it provided youth with a supportive space within which to legitimately explore and express their frustrations with social life. Alongside this, several also demonstrated increased trust and interest in formal political processes. The effects of this medical process on both political consciousness and practices can only partially be attributed to the qualities of the psi therapist or exchange itself. As shown above, the politicization that young women developed throughout their psi experiences also emerged out of the way that psi knowledge and practices derive social meaning in the specific context of the school and within the specific, highly politicized life course of young women themselves.

## Conclusion

The developments in the world of psi practice described in this article are clearly cultivating a permeable and malleable medicalization process. While behavior-based diagnostic categories are being used in a way that generates unease among young people, it is precisely their contested nature that provides an anchor for addressing wider social issues. Ultimately, debates regarding the diagnostic label itself pale in significance compared with the therapeutic process that both psi professional and young patient nurture. Such malleability is allowing psi knowledge to gain greater social prominence, at the same time that it forces therapists to relinquish a certain amount of diagnostic and therapeutic control. By institutionally and ideologically broadening the scope of psi interventions in order to achieve new “democratizing” aims, therapists are simultaneously entrusting teachers, school staff, parents, and young people to use, develop, and modify psi therapy in a relatively organic—if at times conflict-ridden—way (Béhague 2009). That the conflicts that do arise are not consistently dampened by therapy but addressed, and in some cases sustained, signals a potentially important quality of what for some youth eventually became a positive, and even transformational, therapeutic experience.

In giving more attention to the question of how differential forms of medicalization unfold and interact with politicization, this article also highlights an important distinction that must be drawn between psychiatric and social knowledge. In some cases, particularly for young women identified with behavioral disorders, pathways toward medicalization originate and are solidified well before youth come into any meaningful contact with psi professionals themselves. I have shown that reductionistic and alienating forces do not always emanate from forms of medicalization created by psi practitioners, but also from the alienating interactions young people have in the school setting and in society as a whole. The specific role that teachers and school staff play in instigating and sustaining medicalization processes is of critical importance in shaping the extent to which psi therapists are able to implement a form of therapy that touches upon social as well as individual elements. In this way, the successes and failures of the politicizing aspects of therapy can be attributed not to the therapists per se—although they were critical, for they could have insisted on individualizing treatment and reductionistic interpretations, and yet some did not—but rather to the young people themselves and the way they have incorporated the values of what is in effect both a highly medicalized and politicized Brazilian society.

These findings beg the question of which new and unexpected transformations psi professionals may witness in the coming years. For some youth, the very therapeutic process, being a product of modern urban life, was actively sought as a way of learning about modernity, of discussing and debating its effects. The explicit contestation of class- and gender-based norms that psi therapy appears to be enabling also has the potential to challenge wider assumptions in the production of scientific knowledge about the determinants of pathology. Youth are rejecting psi-imbued stereotypes about the nature of their behavioral problems, while at the same time using therapy as a supportive base from which to further what they identify as more acceptable socially imbedded causal explanations for—their difficulties. The creation of these explanations, in turn, appears to be a fundamental stepping stone for the development of politicized practices and problem-solving approaches to life in poverty. If such developments gain greater ground, they hold the potential to unsettle not just highly individualistic biomedical clinical practices, but also some current epidemiological trends that posit the biological, prenatal, and genetic determinants of behavioral disorders as exclusive causes.

The global community of academics and practitioners may have much to learn from the flexibility with which Brazilian psychiatry is practiced. With regard to ADHD in North America and Western Europe, for example, authors have shown that the widespread popular adoption of the category is an outgrowth of the bio medicalization of childhood and motherhood and reflects, in part, a society-wide impetus to “tame” rebellious youth through biologization (Searight and McLaren 1998; Singh 2002a,b; Timimi and Taylor 2004). In Brazil, the process of medicalization, its motivations, and its consequences may be less straightforward. As noted, the degree to which medicalization did or did not nurture greater politicization depended less on clinical factors or the increased use of new behavioral diagnoses, and more on the social conditions in which therapy was sought and unfolded. As therapists confronted entrenched inequities and difficult clinical situations, they maintained an adaptive rather than prescriptive approach; they allowed for, rather than insisted upon, both individualized problem- solving focus and social mobilization. Indeterminacy is well tolerated; indeed, it is encouraged.

At the onset of this article, I insinuated that “ambiguity” has become overused in anthropology and that it is perhaps too imprecise a descriptor of what is happening in some settings, and certainly in parts of Brazil. What I have described in this article is demonstrative not of ambiguity, but of the purposefully equivocal nature of Brazilian medical and social life. Indeed, Plotkin's historical analysis of psychoanalysis in the Southern Cone remarks that, in contrast to Argentinean psychiatrists, Brazilians have always been good at avoiding polarizations and integrating traditions that to outsiders appear conceptually irreconcilable with one another (Plotkin 2001). Yet it would be erroneous to view this as a process of syncretism. Rather, it is a distinctively “hybrid” type of modernity, one that combines, in a “coeval” way, elements from different cultural temporalities—the modern, the premodern, the antimodern, and the amodern (Escobar 1995)—and that allows dissonance to be become an analytic resource (da Costa 1993; DaMatta 1995; Duarte and Guimebelli 1995; Oliven 1996).

The potentially exceptional quality of such developments in Brazil is also instructive for anthropologists of psychiatry more generally. Commitment to understanding how our own intellectual heritage generates assumptions requires us to continually revisit the diverse empirical conditions that render medical practice reductionistic and depoliticized. In this research, for example, I found that the impetus for different forms of medicalization emerge not simply, or even primarily, from within psychiatry, but in the way psi knowledge gains meaning by circulating through—and coming into conflict with—the wider social fabric. The ethnographic reality with which I was confronted also challenged me to move beyond an understanding of everyday lived experience and the construction of illness narratives to consider quite specifically how exceptions to social norms have the potential to modify general social patterns and, indeed, society as a whole. Furthermore, anthropologists have shown how exceptions that deviate from social patterns should not always be understood as acts of “resistance” arising from an explicit and discernable reactionary process. Rather, exceptional social practices arise from ruptures and conflicts within social structures and, thus, hold the potential to become enduring aspects of social life (Fassin 2003; Lock and Kaufert 1998; Lovell 2003). For a minority of youth in Brazil, socially sensitive therapy may be leading to more than just increased political consciousness and activity. Indeed, psi-oriented developments are contributing to the cultivation of a unique group of young community-based leaders committed not simply to the contestation of injustice, but to a form of negotiation based on considerable social dexterity and personal insight. What remains to be seen is whether groups of youth such as these will be able to sustain their positive social roles and, together with therapists, contribute to lasting transformations in their own life course, in psychiatry, and in society as a whole.

## Note

*Acknowledgments*. I am grateful to Professors Margaret Lock and Allan Young for their input and support during the earlier phases of the research upon which this article is based. Many thanks to Helen Gonçalves and Cesar Victor for supporting the anthropological and epidemiological phases of this research. Financial support for different phases of the research was provided by the PanAmerican Health Organization, the U.S. National Science Foundation, the World Health Organization, the Programa Nacional de Núcleos de Excelência (PRONEX-Brazil), the Ministry of Health (Brazil), and the Wellcome Trust. Since 2005, the author has been supported by a postdoctoral fellowship from the Wellcome Trust (Grant no. GR077175MA).
